# O.1.3-4 The MOVING policy index: assessing physical activity policy in 30 European countries

**DOI:** 10.1093/eurpub/ckad133.101

**Published:** 2023-09-11

**Authors:** Jennifer O'Mara, Ioana Vlad, Kate Oldridge-Turner, Arnfinn Helleve, Janetta Harbron, Knut-Inge Klepp, Kate Allen

**Affiliations:** World Cancer Research Fund International, UK; World Cancer Research Fund International, UK; World Cancer Research Fund International, UK; Norwegian Institute for Public Health, Oslo, Norway; University of Cape Town, Cape Town, South Africa; j.omara@wcrf.org; Norwegian Institute for Public Health, Oslo, Norway; World Cancer Research Fund International, UK

## Abstract

**Purpose:**

Physical activity policies play a key role in creating healthier environments where population health and prevention of non-communicable diseases is prioritised. However, fewer than one in five adolescents in Europe meet the daily physical activity recommendations of 60 minutes a day. Greater policy action is needed by national governments to create enabling environments which promote physical activity. The MOVING policy index assesses national government action in physical activity policy across 30 European countries.

**Methods:**

Physical activity policy actions in 30 European countries were identified through a comprehensive scan with a set methodology, including verification by in-country government experts. These policy actions were benchmarked using evidence-informed aspirational attributes that assess the strength of policy design.

**Results:**

*Of the 30 European countries analysed, more than half did not take a comprehensive approach to physical activity policy, implementing policy actions across three key domains: active societies, people and environments. Policy action was concentrated* in public communication, followed by physical activity promotion in schools and the community. Policies that offer physical activity opportunities in the workplace and training in physical activity promotion and that give physical activity training, assessment and counselling in healthcare settings received less attention from governments.

The least action was taken on policy targeting active environments, such as active design guidelines and transport infrastructure, including promoting public and active transport. Eleven of the 30 European countries did not implement any policy actions in either policy area. Countries that did implement policy actions in these areas, received mostly poor assessments for their policy design. For example, 15 out of 22 countries that implemented policy actions on building structures received a poor assessment.

**Conclusions:**

Greater action on better designed policy actions in physical activity is urgently needed across the 30 European countries analysed. Key gaps are seen in policies targeting the built environment and infrastructure for active transport. The MOVING policy index results can be used by policymakers, researchers and civil society, both at European or country level to inform advocacy for and design of physical activity policies.

